# Previously Undiagnosed Spinal and Bulbar Muscular Atrophy as a Cause of Airway Obstruction after Robot-Assisted Laparoscopic Prostatectomy

**DOI:** 10.1155/2017/9780265

**Published:** 2017-07-17

**Authors:** Miyuki Niki, Taihei Tachikawa, Yuka Sano, Hiroki Miyawaki, Aisa Matoi, Yukari Okano, Nobutaka Kariya, Tsuneo Tatara, Munetaka Hirose

**Affiliations:** ^1^Department of Anesthesiology and Pain Medicine, Hyogo College of Medicine, Nishinomiya, Hyogo, Japan; ^2^Department of Anesthesia, Meiwa Hospital, Nishinomiya, Hyogo, Japan; ^3^Intensive Care Unit, Hyogo College of Medicine Hospital, Nishinomiya, Hyogo, Japan

## Abstract

**Background:**

Preoperative vocal cord paralysis is a risk factor for postoperative respiratory distress following extubation after general anesthesia. We present an unusual case where a geriatric patient developed airway obstruction after robot-assisted laparoscopic prostatectomy.

**Case Presentation:**

A 67-year-old male, who had suffered from left vocal cord paralysis of unknown etiology, was scheduled for robot-assisted laparoscopic prostatectomy (RALP). General anesthesia was performed without any problems. The patient, however, developed airway obstruction one hour after extubation and was reintubated following commencement of mechanical ventilation for one day. At the age of 70 years, the patient received an emergency tracheostomy due to bilateral vocal cord paralysis and then was diagnosed with spinal and bulbar muscular atrophy (SBMA). Although no muscle weakness of either upper or lower extremities was observed, rocuronium showed hypersensitivity during total laryngectomy under general anesthesia.

**Conclusions:**

Vocal cord paralysis combined with postoperative laryngeal edema, the cause of which was presumed to be SBMA, likely caused airway obstruction after RALP. As neuromuscular symptoms progress gradually in patients with SBMA, muscle relaxants should be used carefully, even if patients with SBMA present no immobility of their extremities.

## 1. Background

A 6-degree head-down tilt position during surgery induces fluid shift from the lower to the upper body, along with cardiopulmonary baroreceptor reflex responses including bradycardia, an increase in urinary excretion of sodium, and thermoregulatory changes [1, 2]. In addition to these physiological responses, a steep 25–40-degree head-down tilt position during robot-assisted laparoscopic prostatectomy (RALP) may cause postoperative laryngeal edema [3]. Severe laryngeal edema requiring reintubation after RALP, however, occurs rarely and the reason why it occurs only rarely is uncertain [4].

On the other hand, spinal and bulbar muscular atrophy (SBMA), also known as Kennedy's disease or Kennedy-Alter-Sung disease, is a rare neurodegenerative disorder of lower motor neurons with an X-linked recessive inheritance pattern and occurs virtually only in adult males [5–7]. There are several potential anesthetic risk factors, including laryngospasm, hyperkalemia with use of depolarizing muscle relaxants, increased sensitivity to nondepolarizing muscle relaxants, and postoperative respiratory failure or aspiration [7].

We report a case of a male patient who was reintubated due to airway obstruction after undergoing general anesthesia for RALP. He was subsequently diagnosed with SBM three years later. The patient provided written permission for the authors to publish this report.

## 2. Case Presentation

A 67-year-old male, ASA physical status II, diagnosed with prostatic adenocarcinoma was scheduled for RALP under general anesthesia. He had suffered from left vocal cord paralysis of unknown etiology since the age of 50 years. He had also been diagnosed with adenomatous goiter at the age of 56.

After assessment by the anesthesiologists, it was decided that despite the steep head-down tilt position the patient would tolerate general anesthesia, using a tracheal tube with a smaller diameter. General anesthesia was induced with 100 mg of propofol, 50 *μ*g of fentanyl, and 50 mg of rocuronium intravenously and was maintained with 600 *μ*g of fentanyl, intravenous continuous infusion of remifentanil (0.1–0.4 *μ*g·kg^−1^·min^−1^), sevoflurane (end-tidal: 1.0–1.5%), and rocuronium. Intravenous rocuronium was injected intermittently following the simulated effect-site concentration of rocuronium during surgery.

Following induction, the patient was intubated under direct laryngoscopy with a cuffed tracheal tube, ID 6.5 mm, after observing that there were no laryngeal or pharyngeal abnormalities besides the left vocal cord paralysis via a fiber-optic bronchofiberscope. There were no intraoperative problems with surgery or anesthesia.

After surgery concluded, intravenous 120 mg sugammadex (2 mg/ideal body weight) was administered for reversal of rocuronium. The patient was extubated without any problems with the Aldrete score of 10 and transferred to our intensive care unit (ICU) for routine observation after RALP. Surgical time was 6 h and 31 min and anesthetic time was 8 h 36 min. Total amount of crystalloid infused was 2950 mL, intraoperative urine output was 740 mL, and intraoperative blood loss was 300 mL.

In the intensive care unit, the patient developed dyspnea with paradoxical respiration about one hour after extubation. Fiber-optic bronchofiberscope examination revealed airway obstruction due to laryngeal edema with vocal cord paralysis. Midazolam was injected intravenously, and the patient was reintubated with an uncuffed tracheal tube, ID 5.0 mm, after we were unable to pass an ID 5.5 mm uncuffed tracheal tube through the edematous larynx. The patient was positioned in head-up tilt, and intravenous methylprednisolone was administered following commencement of mechanical ventilation. The patient was successfully extubated 12 h after reintubation and reported no dyspnea thereafter. He was discharged from ICU 3 days after the operation.

At the age of 68, the patient underwent left thyroidectomy due to adenomatous goiter. Intravenous 70 mg of propofol and 100 *μ*g of fentanyl were administered for induction of general anesthesia, and 50 mg of rocuronium was used to facilitate tracheal intubation with a 6.5 mm ID endotracheal tube. Surgery proceeded and general anesthesia was maintained with sevoflurane (end-tidal: 1.0–1.5%), 100 *μ*g of fentanyl, intravenous continuous infusion of remifentanil (0.1–0.3 *μ*g·kg^−1^·min^−1^), and 100 mg of rocuronium. There were no intraoperative problems. At the end of the procedure, reversal of muscle relaxant was achieved using 120 mg of sugammadex. Video-laryngoscopy confirmed that there was no laryngeal edema present. The tracheal tube was removed without any problems. Total surgical time was 1 h 11 min, and anesthetic time was 2 h 8 min.

At the age of 69 years, the patient underwent uneventful inguinal hernia repair under spinal anesthesia, using 3.2 ml of 0.5% bupivacaine. The highest level of spinal block achieved was Th8.

**Figure 1:**
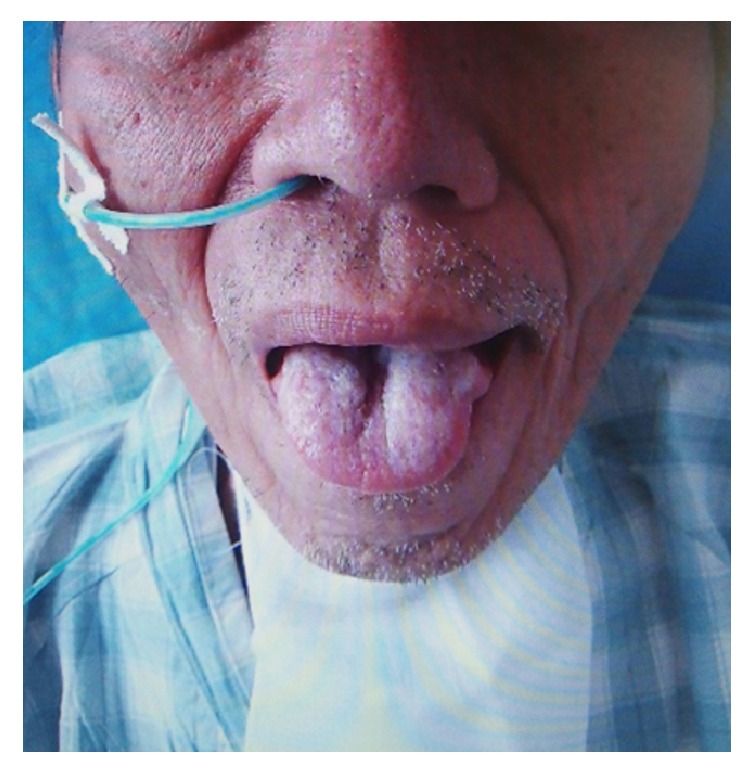
Atrophy and fasciculation of the tongue at the time of diagnosis of spinal and bulbar muscular atrophy (SBMA).

At the age of 70 years, the patient suffered from dysphagia and dyspnea suddenly with the development of pyrexia within one month. An emergency tracheostomy was performed under local anesthesia after bilateral vocal cord paralysis was identified. Clinical examination revealed bulbar paralysis with atrophy and fasciculation of the patient's tongue (Figure 1).

Although the patient had no muscle weakness of either upper or lower extremities, deep tendon reflexes were suppressed. After genetic testing revealed an expanded CAG repeat, the number of which was 45 times, in the androgen receptor (AR) gene, he was diagnosed with SBMA. The patient did not have gynecomastia, testicular atrophy, glucose intolerance, or hyperlipidemia, Brugada syndrome, or a high level of plasma creatine kinase; these findings occasionally accompany SBMA.

Total laryngectomy was scheduled after he was diagnosed as having SBMA. His tracheostomy tube was replaced to an armoured tracheal tube under general anesthesia using 6.0% end-tidal concentration of desflurane and 50 *μ*g of fentanyl. Train-of-four ratio (TOFR), which was measured by stimulating the left ulnar nerve using TOF-Watch (Organon, Dublin, Ireland), was 1.0 before rocuronium injection. Two min after the injection of 30 mg of rocuronium (0.6 mg/ideal body weight), TOFR reached 0. General anesthesia was maintained with desflurane (end-tidal: 4.0–4.5%), 100 *μ*g of fentanyl, and intravenous continuous infusion of remifentanil (0.1–0.3 *μ*g·kg^−1^·min^−1^). Recovery of the first twitch height (T1) of TOFR was delayed at 100 min after rocuronium injection. T2 and T4 were obtained at 150 min and 180 min, respectively. Although 110 mg of sugammadex (2 mg/ideal body weight) was administered at the end of surgery, TOFR remained at 0.5. After we administered the same dose of sugammadex additionally, TOFR recovered to 0.67. We extubated a tracheostomy tube, and his Aldrete score was 10 at the end of anesthesia. The duration of surgery was 2 h 21 min, and the duration of anesthesia was 3 h 39 min. There were no problems in the ICU, and the patient was discharged from ICU the next day of surgery.

## 3. Discussion

SMBA is characterized by progressive weakness in the lower and upper extremities, bulbar weakness, laryngospasm, gynecomastia, and tremor and occurs only in adult males [5–7]. Some clinical features of SMBA resemble the early stages of amyotrophic lateral sclerosis. SMBA is caused by the enlargement of a CAG repeat in the AR gene on the X chromosome, which encodes glutamine tract in ARs. The number of CAG repeats over 38 is diagnostically useful and is often associated with androgen insensitivity [8]. Its estimated prevalence is 1-2 per 100,000 [9]. Suggested pathophysiological mechanisms of neurodegeneration involve formation of nuclear inclusions by the polyglutamine-expanded AR (polyQ AR) and/or release of toxic soluble form of oligomerized polyQ AR and alterations of the ubiquitin-proteasome system and of autophagy pathways [10] Neuronal and muscular dysfunction may be a consequence of loss of normal functions of AR and/or gain of functions of toxic form of polyQ ARs which accumulate in motor neurons [10].

Given that SMBA is a motor neuron disease with bulbar dysfunction, many anesthesiologists may be concerned about the use of neuromuscular blocking drugs and their reversal agents. Interestingly, however, there have been no reported complications following the use of these agents so far [7, 11]. In this case, neither rocuronium nor sugammadex, used during the first and second instances of general anesthesia, was associated with any complications when the patient was 67 and 68 years old. In the third instance of general anesthesia at the age of 70 after the patient was diagnosed with SBMA, however, rocuronium showed hypersensitivity because of the prolonged neuromuscular blocking effect of rocuronium with insufficient recovery of TOFR after the administration of sugammadex [12]. Although he had no muscle weakness at the extremities, dysfunction of motor neurons might cause this hypersensitivity to nondepolarizing muscle relaxant. If a patient shows immobilization at the extremities, anesthesiologists need to take their anesthetic management into consideration that depolarizing muscle relaxant could induce hyperkalemia, as immobilization upregulates immature types of nicotinic acetylcholine receptors at the skeletal muscle [13]. As neuromuscular symptoms progress gradually in patients with SBMA, muscle relaxants should be used carefully, even if patients with SBMA present no immobility of their extremities.

Niesen et al. examined the anesthetic records of six patients with SBMA who underwent general anesthesia and identified one 66-year-old patient who developed severe postoperative glottic edema after surgery for a tibial fracture [7]. Although this patient had proximal muscle weakness and bulbar dysfunction preoperatively, the cause of glottic edema was unclear.

Androgens inhibit activation of complement by enhancing C1-esterase inhibitor [14]. In hereditary angioneurotic edema, activation of the classical complement pathway, caused by the deficiency of C1-esterase inhibitor, induces laryngeal edema, and androgens have been used for treatment in this disease [15].

As such, it is speculated that, in our present case, activation of the classical complement pathway due to androgen insensitivity in SBMA likely augmented his laryngeal edema after the steep head-down tilt position was employed during RALP. Before this procedure, he had existing left vocal cord paralysis, which may have been caused by the loss of lower motor neurons supplying the laryngeal muscles. In view of this, it is our opinion that vocal cord paralysis secondary to SBMA, combined with postoperative laryngeal edema, likely caused airway obstruction after RALP.

## 4. Conclusion

In this patient, preoperative vocal cord paralysis combined with laryngeal edema, which was facilitated by a steep head-down tilt position during surgery, caused airway obstruction after extubation. SBMA was diagnosed three years later and is presumed to be the cause of the patient's bulbar symptomatology. As motor dysfunction progresses slowly in SBMA patients, anesthetic management needs to be carefully tailored, paying particular condition to the clinical presentation.
